# Pure and Doped Brushite Cements Loaded with Piroxicam for Prolonged and Constant Drug Release

**DOI:** 10.3390/ma18051065

**Published:** 2025-02-27

**Authors:** Marcella Bini, Giovanna Bruni, Michela Sturini, Beatrice Rossetti, Gianluca Alaimo, Ferdinando Auricchio, Valeria Friuli, Lauretta Maggi

**Affiliations:** 1 Chemistry Department, University of Pavia, Viale Taramelli 16, 27100 Pavia, Italy; giovanna.bruni@unipv.it (G.B.); michela.sturini@unipv.it (M.S.); 2 Consorzio per i Sistemi a Grande Interfase (CSGI), Via Della Lastruccia 3, 50019 Sesto Fiorentino, Italy; 3 Department of Civil Engineering & Architecture, University of Pavia, Via Ferrata 3, 27100 Pavia, Italy; beatrice.rossetti@unipv.it (B.R.); gianluca.alaimo@unipv.it (G.A.); ferdinando.auricchio@unipv.it (F.A.); 4 Department of Drug Sciences, University of Pavia, Viale Taramelli 12, 27100 Pavia, Italy; lauretta.maggi@unipv.it

**Keywords:** calcium phosphate cements, brushite phase, doping, mechanical measurements, piroxicam, dissolution rate

## Abstract

The increase in life expectancy has led to a rise of musculoskeletal disorders. Calcium phosphate cements (CPCs), thanks to some amazing features such as the ability to harden in vivo, bioactivity, and resorbability, are promising candidates to treat these diseases, notwithstanding their poor mechanical properties. We aimed to synthesise pure and barium- or silicon-doped brushite-based CPCs loaded with piroxicam to study the effects of the substitution on physical-chemical and pharmaceutical properties before and after cement immersion in phosphate buffer for different time periods. Our results demonstrated that piroxicam became amorphous in the hardened cements. The dopants did not change the brushite structure or its lamellar morphology, while both Ba and Si additions improved the initial Young’s modulus compared to the pure cement, and the opposite trend was observed for compressive strength. Both the compressive strength and the elastic modulus decreased for the samples immersed in solution compared to the non-immersed samples, with stabilisation as the number of days increased. After 7 days, the whole drug amount was released, with a slower and constant kinetic for the Ba-doped cements compared to the pure and Si-doped ones.

## 1. Introduction

The increased life expectancy in the developed countries of the world inevitably led to the growth of musculoskeletal disorders, such as osteoporosis and osteoarthritis. In the meantime, new care and drugs to treat these pathologies are being discovered [1,2]. However, the main issue related to the use of new drugs is the need to target and control the drug release to a specific body site, so many efforts are devoted to developing materials suitable for targeted drug delivery [3,4]. In this context, calcium phosphate cements (CPCs) seem particularly promising due to many advantages, mainly the ability to harden in vivo through a low-temperature setting reaction. In fact, CPCs are hydraulic cements, formed by mixing more than one calcium orthophosphate with a liquid phase: a mouldable paste is obtained, which can be injected during surgery using minimally invasive procedures [5,6]. Due to the non-exothermic character of the setting reaction of CPCs, the incorporation of different drugs and biological molecules is possible, making them good candidates for bone repair but also for drug delivery applications [7,8], being the drug host in the entire cement volume so allowing its prolonged release. CPCs have excellent bioactivity, the ability to form direct bonds with bone, osteoconductivity, and resorbability [9,10].

CPCs can find applications in many different fields mainly thanks to their injectability, which can be improved for example by decreasing the powder to liquid ratio during cement formulation, increasing the viscosity of the liquid phase, reducing the particle interactions and the particle sizes of the powder, and dissolving sodium citrate or citric acid in the cement liquid phase. Thanks to the injectability allowing minimally invasive surgical procedures, the CPCs can be exploited in orthopaedics, e.g., to fill bone defects due to trauma or tumours and to stabilise fractures. CPCs were particularly useful for treating defects in the proximity of joints, mainly in older patients or even solving craniotomy defects in several animal models. Other important applications can be found in oral and maxillofacial surgery for vertical bone augmentation and bone defect healing. In these cases, CPCs can be used in the form of injectable cements or as preset cement granules. CPCs can regenerate bone in atrophic areas, buccal dehiscence defects, and maxillary sinuses and promote bone healing in post-extraction dental alveolar sockets. [11,12,13,14,15,16].

However, CPCs also have some drawbacks, mainly poor mechanical performances, which limit their applicability to moderate load-bearing conditions, also due to their intrinsic porosity [11]. The cement features also depend on the phase that is stabilised after the setting reaction on the base of composition but also pH, namely, hydroxyapatite (HAP) or brushite (dicalcium phosphate dihydrate, DCPD) [7,17]. In particular, Ca-deficient HAP, formed because of Ca^2+^ loss from HAP, can be found in bone minerals. If compared to stoichiometric HAP, it has a greater specific surface area and higher resorbability and solubility in water and body fluids. It is also well known that the chemical composition and crystallite sizes of synthetic HAP can be easily tailored by varying the experimental conditions of the synthesis [18]. Ca-deficient HAP can generate an apatite layer on its surface that resembles bone morphology, favouring osteointegration. One of its main uses is in implant coatings. Brushite, differently from HAP, is metastable under physiological conditions, and, for this reason, brushite CPCs resorb faster than apatitic ones, although it has been shown that DCPD can convert in vivo into HAP [19]. The brushite CPCs are obtained as a result of an acid–base reaction and they have better mechanical properties with respect to the HAP-based CPCs and for this reason are the most diffused ones.

A strategy to improve the small compressive strength values of brushite CPCs is the doping with different elements replacing Ca or P (such as Mg^2+^, Sr^2+^, Zn^2+^, Si^4+^, V^5+^) in the brushite unit cell [20,21,22,23,24,25,26,27]. The main aim of the substitution is certainly to improve the mechanical properties of the cements, but also to allow the release of ions into the body that could be beneficial for peculiar biological processes. Another promising strategy to improve the poor mechanical properties and develop synthetic bone substitutes is the biomimetic approach, in which a brittle ceramic material is coupled with an elastic polymer to mimic the composite structure of bone tissue, particularly for vertebroplasty or oral applications [15,28].

For what concern dopants, a controversial substituent for biomaterials is certainly the Ba^2+^ ion, because it has been reported for a long time that this ion is toxic to the body. However, at present, no sufficient studies have been devoted to deepening its in vivo toxicity, which seems confirmed in cases of oral or intravenous administration, but when barium was combined with calcium phosphates, such as HAP or α-Ca_3_(PO_4_)_2_, the resulting cements exhibited nontoxicity and biocompatibility, with faster setting time [29]. Moreover, it was reported that by increasing the Ba^2+^ amount, the ability to form apatite also increased.

Silicon instead, when used as a substituent, can help to increase the bone mineral density and improve the cartilage synthesis, the vasculogenesis, and the bone mechanical properties [30,31].

Our aim was to synthesise pure and Ba- or Si-doped brushite-based cements, loaded with Piroxicam, a poorly water-soluble nonsteroidal anti-inflammatory drug (NSAID).

Silicon was mainly chosen because it was reported that it could improve the mechanical properties and bone density of CPCs, while barium, notwithstanding distrust, could help in promoting hydroxyapatite formation, improving the osteogenesis and possibly mechanical properties.

The cements were prepared by mixing the drug and calcium orthophosphate powders with the liquid phase. The effect of the substituents on the brushite crystal structure and morphology was investigated with the combined use of different physical-chemical techniques, such as X-Ray powder diffraction (XRD), Fourier transform infrared spectroscopy (FT-IR), scanning electron microscopy (SEM) with energy dispersive spectroscopy (EDS), and inductively coupled plasma optical emission spectroscopy (ICP-OES). These techniques were applied to first characterise the pure and doped β-tricalcium phosphate, a cement precursor, and then the cements before and after the immersion in phosphate buffer. The mechanical properties of cements, i.e., compressive strength and Young’s modulus, and the dissolution rate of drug were analysed in view of the substituent presence and the time of immersion in phosphate buffer (up to 7 days).

The novelty of this paper is related to the preparation of new doped brushite cements loaded with the anti-inflammatory piroxicam, never used with brushite cements, to sustain a prolonged and targeted release in the first days after the surgery, for a possible use in orthopaedic implants.

## 2. Materials and Methods

**Scheme 1:**
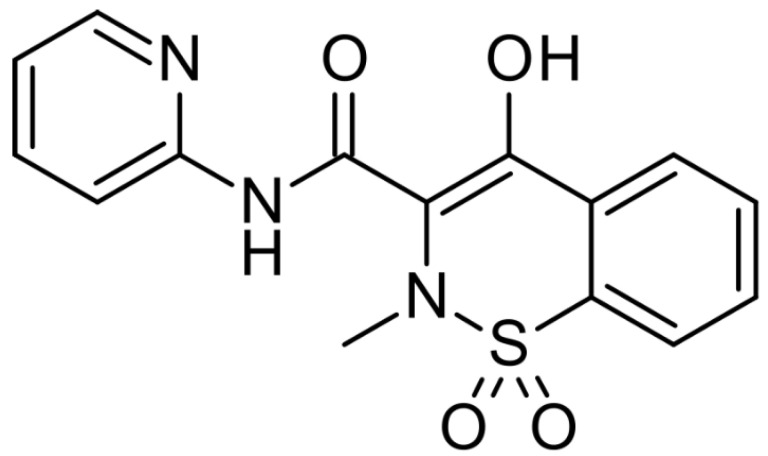
Molecular structure of piroxicam.

All the chemicals employed for the β-tricalcium phosphate (β-TCP, β-Ca_3_(PO_4_)_2_), one of the cement precursors, syntheses were of reagent grade and were purchased from Sigma-Aldrich (Sigma-Aldrich, St. Louis, MO, USA). Monocalcium phosphate monohydrate Ca(H_2_PO_4_)_2_ H_2_O (MCPM) was purchased from Santa Cruz Biotechnology (Santa Cruz Biotechnology, Dallas, TX, USA), while piroxicam (Prx) (Scheme 1) was kindly donated by Olon (Olon, Casaletto Lodigiano, Italy).

### 2.1. Synthesis of Pure and Doped β-TCP

β-TCP (the β polymorph of Ca_3_(PO_4_)_2_) was prepared by a common solid-state synthesis [32] starting from CaCO_3_ and CaHPO_4_ 2H_2_O (1:2 stoichiometric ratio) following the reaction

CaCO_3_ + 2CaHPO_4_ 2H_2_O →Ca_3_(PO_4_)_2_ + CO_2_ +3H_2_O (1)

The reagents were mixed in a mortar, then transferred in an alumina boat and treated in an oven in air at 1000 °C for 15 h. This sample was named β-TCP.

Two doped β-TCP, Ca_2.9_Ba_0.1_(PO_4_)_2_ and Ca_3_(PO_4_)_1.95_(SiO_4_)_0.05_, were also prepared. The amount of dopant was chosen, inspired by the recent literature [23,24,25] and, for silicon, because a higher amount of dopant produced secondary phases (see also the Discussion section for more details). For Ba doping, CaCO_3_ was partially substituted by BaCO_3_, while for Si doping, some DCPD was substituted by CaSiO_3_, as a silicon source, following the reaction (1).

The reagents were mixed in a mortar and the syntheses proceeded as for the pure β-TCP in the case of the Ba-doped sample, while for the Si-doped sample, the thermal treatment was performed at 1100 °C for 15 h. These samples were named β-TCP-Ba and β-TCP-Si.

### 2.2. Synthesis of Cements

The cements were prepared starting from pure or doped β-TCP and MCPM, following the reaction (for the pure cement as an example):

Ca_3_(PO_4_)_2_ + Ca(H_2_PO_4_)_2_ H_2_O + 7H_2_O → 4CaHPO_4_ 2H_2_O (2)

The same amount of the two reagents was weighted and then mixed in a mortar. A citric acid solution (0.5 M) was added in the ratio 1 mL/2 g (liquid/powder ratio), and the obtained paste was transferred into a plastic mould (7 mm height, 7 mm diameter, according to the ASTM F451-08 standard used as a reference). The cement cylinders were allowed to set in an oven at 25 °C for 24 h. These samples were named CEM, CEM-Ba, and CEM-Si.

The procedure to prepare the active principle loaded cements was the same, apart from the addition to the solid mixture of phosphates (reaction 2) of a 5 wt% amount of piroxicam (with respect to MCPM). These samples were named CEM-Prx, CEM-Ba-Prx, and CEM-Si-Prx.

### 2.3. Physical-Chemical Characterisation

For the X-Ray powder diffraction (XRD) measurements, a Bruker D5005 diffractometer (Bruker BioSpin, Fällanden, Switzerland) with Cu Kα radiation, a graphite monochromator, and a scintillation detector was employed. The patterns were collected in the air with the following conditions: a step size of 0.03° and a counting time of 2 s/step in the 10–70° angular range using a silicon sample holder.

The Fourier transformed infrared (FT-IR) spectra were collected using a Nicolet FT-IR iS20 spectrometer (Nicolet, Madison, WI, USA) with the ATR (attenuated total reflectance) sampling accessory (Smart iTR with diamond plate). The spectra were obtained by co-adding 32 scans in the 4000–600 cm^−1^ range at a 4 cm^−1^ resolution.

A scanning electron microscope (SEM) (Zeiss Evo MA10; Carl Zeiss, Oberkochen, Germany) coupled with an energy-dispersive spectroscopy (EDS) detector for microanalysis (X-max 50 mm, Oxford Instruments, Wiesbaden, Germany), working at an acceleration voltage of the electron beam of 20 kV, was used to collect the images and microanalysis spectra. For SEM analysis, the samples were sputtered with gold, while for EDS, they were used without metallic coating.

Ca, Si, and P measurements were performed with a Thermo-Fisher iCAP 7400 ICP-OES (Thermo Fischer Scientific, Waltham, MA, USA) equipped with a cyclonic spray chamber, a concentric nebuliser, and a ceramic duo-torch. Weighted amounts of each sample were treated with a few millilitres of ultrapure 65% HNO_3_ and 37% HCl (2:1 mL), refluxed for 15 min, evaporated to a small volume, and diluted to 40 mL with ultrapure water. The clear solution thereby obtained was properly diluted (1:10 or 1:5) before ICP-OES measurements.

### 2.4. Pharmaceutical Characterisation: Dissolution Test and Statistical Analysis

An in vitro dissolution test was designed to verify the release profile of Prx from the cements, all containing 9 mg of the drug. The USP apparatus 1, basket, was used [33] (Erweka DT-D6, Erweka GmbH, Dusseldorf, Germany), which includes six glass containers with a cylindrical shape and a hemispherical bottom on which a lid is placed to avoid evaporation of the dissolution medium, that is, 500 mL of isotonic phosphate buffer at pH 7.4. The vessels were contained in a bath thermostated at 37 ± 0.5 °C. The dissolution fluid was normally at rest; the rotation of the baskets at 30 rpm was activated every 3 h for 15 min. At pre-set time intervals, a pump automatically withdrew a portion of the solution, sent it to the flow cells of the spectrophotometer (Lambda 25, PerkinElmer, Monza, Italy) and finally re-injected it into the vessel from which it was taken. The absorbance values were processed, via a calibration line previously carried out, by a specific software (Winlab V6 software, PerkinElmer, Monza, Italy). Readings of the drug amount were taken on days 1, 4, and 7 (on three samples each), and then the cylinders were recovered and placed in an oven at 50 °C under vacuum to facilitate the evaporation of the fluid absorbed during the dissolution tests until constant weight.

The statistical evaluation of similarity factor *f*_2_ is a criterion to assess whether two dissolution curves can be considered similar or different and is calculated using the following equation [34]:

$$f_{2}=50\times log\left\{{\left[1+\left(1/n\right)\sum_{t=1}^{n}{\left(R_{t}-T_{t}\right)}^{2}\right]}^{0.5}\times 100\right\}$$

where *R_t_* and *T_t_* are the cumulative percentage dissolved at each point of sampling time (*n*) of the reference and the tested profile, respectively.

When the calculated *f*_2_ value between two dissolution curves was between 50 and 100, the two phenomena could be considered similar, but if it was less than 50, the two profiles could be regarded as different.

### 2.5. Pharmaceutical Characterisation: Dissolution Kinetics Analysis

Different models can be used to interpret the drug release kinetics for this type of system; however, some of them can only be applied by respecting strict boundary conditions [35]. So, we tested zero-order and first-order kinetics fitting, while, in our experiments, the boundary conditions were not satisfied for the Higuchi, Korsmeyer–Peppas, and Hixson–Crowell models [36].

A zero-order kinetics was obtained when the drug release rate was quite constant from pharmaceutical forms, and thus the drug concentration at the site of action was able to be maintained constant as well [37]. The slope of the graph obtained by plotting the percentage of drug dissolved against time (hours) gives the zero-order rate constant (3).

C_0_ − C_t_ = K_0t_ (3)

where C_t_ is the amount of drug dissolved at time t, C_0_ is the concentration of drug at time t = 0, and K_0t_ is the zero-order rate constant.

A first-order kinetics model can better describe the drug release mechanism when the release rate is directly proportional to the drug concentration, and thus the release rate is not constant during the process but much faster at the beginning and much slower in the final phase of the dissolution test. The log of the percentage of the drug remaining in the sample is plotted against time (hours). The slope gives the first-order rate [38].

log C = log C_0_ − K_1t_/2.303 (4)

where K_1t_ is the first-order rate equation expressed in time or per hour, C_0_ is the initial concentration of the drug, and C is the percent of drug remaining in the sample at time t.

### 2.6. Mechanical Characterisation: Compressive Test

To perform the compressive test, an MTS Insight machine with a twin-column tabletop model and a 10 kN load cell was used (Figure S1). Displacements were measured through the crosshead displacement sensor integrated into the testing machine.

The tests were carried out on cylindrical specimens with an average diameter of 7 mm and an average height of 7 mm as a result of an adjustment upon removal from the mould, since the samples exhibited minor surface defects, which required corrective post-processing. The deformation rate chosen was equal to 1 mm/min. The load–displacement experimental data were used to calculate both the compressive strength and the Young’s modulus values, where the compressive strength is the stress corresponding to the peak force recorded. The target number of specimens tested was at least 5 for each type of sample (regarding both the type of cement and the drug release days). However, because of test non-conformities such as premature damage to the specimen and defects that led to failure immediately after the start of the test, both due to the level of porosity of the cylinders, the actual number of samples tested was as reported in Table S1.

### 2.7. Statistical Analysis

From the mechanical tests, the key parameters extracted were the compressive strength [MPa] and the Young’s modulus [MPa]. The compressive strength was defined as the ultimate load at failure divided by the initial cross-sectional area of the specimen.

To ensure the reliability of the data, a quartile analysis was performed to detect and exclude outliers. For each series, the first (Q1) and third (Q3) quartiles were calculated, and any data points falling inside the range [Q1 − 1.5 (Q3 − Q1), Q3 + 1.5(Q3 − Q1)] were accepted, whereas data points falling outside were considered outliers and removed from the dataset. After the quartile analysis, the arithmetic mean and standard deviation (SD) of the accepted points were computed. The SD was calculated using the formula:

$$\sqrt{\frac{1}{N}\;\sum_{i=1}^{n}(x_{i}-\bar{x}{)}^{2}}$$

where *N* is the number of measurements, $x_{i}$ are the individual data points, and $\bar{x}$ is the mean.

The values of compressive strength and Young modulus, presented in the Results, Section 3.3, corresponded to the mean values, with error bars representing the standard deviation.

## 3. Results

### 3.1. Physical-Chemical Results

The physical-chemical characterisation was performed, at first, for the pure and doped β-TCP samples by using XRD, FT-IR, SEM/EDS, and ICP-OES data to verify the phase formation, chemical composition, and morphologies. Then, the same combined analysis was applied to the prepared cements.

#### 3.1.1. Pure and Doped β-TCP

**Figure 1:**
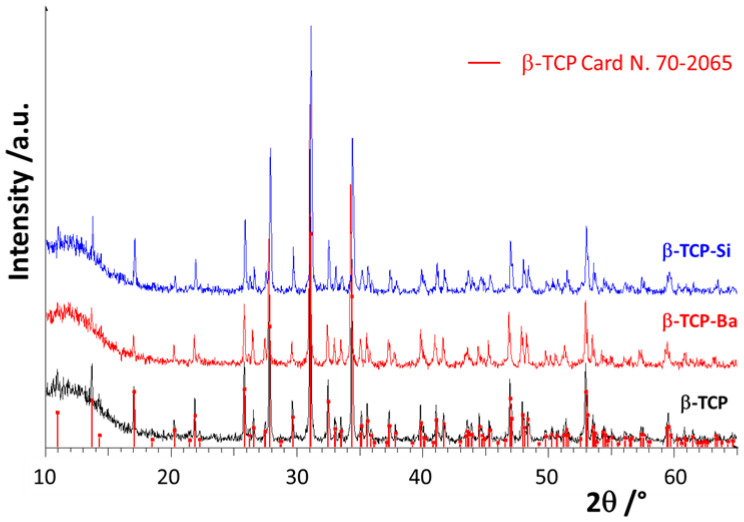
XRD patterns of β-TCP, β-TCP-Ba, and β-TCP-Si. The bars of the expected peak positions of β-Ca_3_(PO_4_)_2_ (red) are also reported.

The β-TCP phases were prepared as described in the Section 2.1 and Section 2.2. First, it should be verified if the dopants can be hosted in the β-TCP structure, even if in the literature there are many successful examples of doping with, for example, Mg^2+^, Sr^2+^, and Zn^2+^ [23,24,25]. It is reported that the β polymorph of Ca_3_(PO_4_)_2_ is stable up to 1125 °C, and then it transforms into the α phase [39]. It has a rhombohedral structure (space group R3c), with Ca located on five distinct sites with coordination numbers varying from 3 to 8. The threefold coordinated site has a partial occupation of calcium, as demonstrated by neutron diffraction [40]. Phosphorous ions are located on three different tetrahedral sites having similar bond lengths. The XRD patterns of pure and doped β-TCP are reported in Figure 1 and compared with the bars referring to the expected peak positions of the calcium phosphate. The patterns are similar, and the peak positions and intensities agree well with the bars. This suggests that the β-TCPs are pure phases, and the dopants do not introduce structural variations, nor segregate as impurity phases. Some small differences in intensities ratios could be due to small variations of structure factors because of cation substitution, as well as to small differences in the sample’s crystallinity.

**Figure 2:**
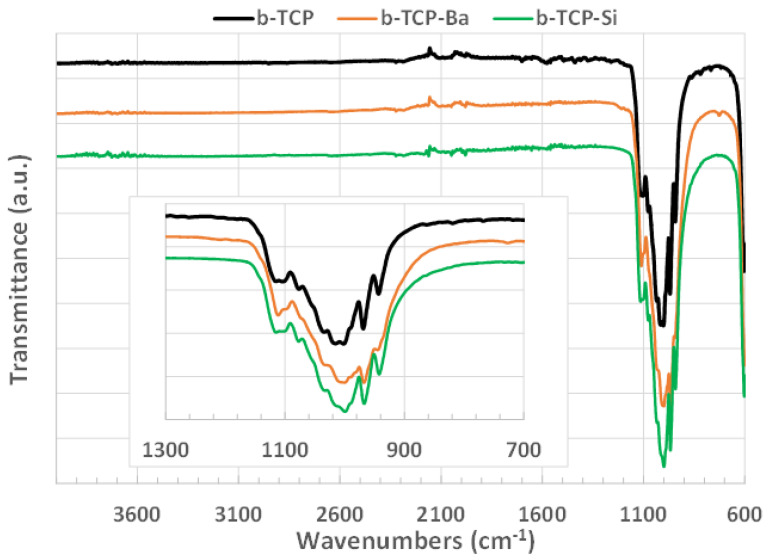
FT-IR spectra of β-TCP, β-TCP-Ba, and β-TCP-Si in the 4000–600 cm^−1^ spectral range. In the inset, the enlargement of the 1300–700 cm^−1^ spectral range is shown.

To further verify that the dopants do not introduce structural variations in β-TCP, FT-IR spectra were also collected (Figure 2).

It is evident that the spectra were similar and showed the same main peaks at around 1000 cm^−1^, characteristics of phosphates. In fact, as reported in the literature [41,42], the free phosphate PO_4_^3−^ ion, with an ideal T_d_ point symmetry, has four modes of vibration: the antisymmetric and symmetric stretching at about 1082 cm^−1^ and 980 cm^−1^ and the antisymmetric and symmetric bending at about 567 cm^−1^ and 420 cm^−1^, respectively.

However, the band multiplicities (Figure 2) show that the local symmetry of the PO_4_^3−^ group in β-TCP was reduced from T_d_ to C_1_. In addition, three independent PO_4_^3−^ groups were present in the structure, thus increasing the number of observable bands. In fact, at least six intense peaks can be resolved in the antisymmetric 1200–1000 cm^−1^ stretching range. In particular, for the pure β-TCP, peaks at about 1126, 1101, 1075, 1047, 1013, and 1000 cm^−1^ can be detected (Figure 2, black curve). By enlarging the 1300–700 cm^−1^ spectral range (Figure 2, inset), it can be noted that the peaks of β-TCP-Ba, at least those at about 968 and 942 cm^−1^, were broadened, while that at about 1120 cm^−1^ seemed more defined with respect to the β-TCP and β-TCP-Si. These pieces of evidences can be due to the structural disorder introduced by Ba substitution in the crystal structure, differently from the Si substitution, which did not produce evident changes, probably due to the similarity of PO_4_^3−^ and SiO_4_^4−^ tetrahedral units. Some shifts of peaks were also present, in particular for the main peak at about 1000 cm^−1^, which slightly shifted to lower wavenumbers passing from pure β-TCP to Ba- and Si-doped samples. Similar effects are reported in the literature for other doped β-TCP samples [24,25].

**Figure 3:**
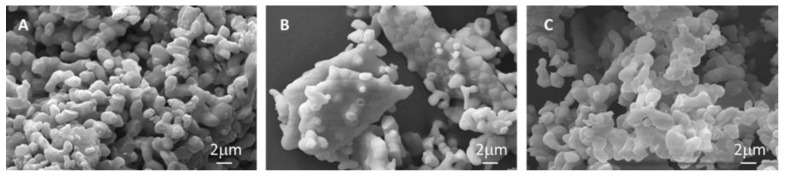
SEM images of β-TCP (**A**), β-TCP-Ba (**B**), and β-TCP-Si (**C**).

The morphology of the β-TCP samples is shown in Figure 3.

The pure sample (Figure 3A) was constituted by rounded grains that, in some parts, seem fused together, similar to analogous samples reported in the literature [24,25]. Some particles had cut edges, obviously formed during the grain growth at high temperatures. In both the doped samples, a higher aggregation with respect to the pure sample was evident. In the β-TCP-Ba sample (Figure 3B), the grains were linked together, even if some grain boundaries were evident. The β-TCP-Si was more likely the pure one, even if the grains were blunt and less defined (Figure 3C).

EDS and ICP-OES analyses, employed to verify the Ca/P ratio and the dopant amount, completed the physical-chemical characterisation. We can remember that the value of the Ca/P stoichiometric ratio for the Ca_3_(PO_4_)_2_ phase was 1.5, while for the β-TCP-Ba and β-TCP-Si, it should be 1.45 and 1.54, based on the corresponding chemical formula (see Section 2.2). The Ca/P ratio obtained by EDS analysis (Table 1) compared well with the expected values within the error of the measurement.

For what concerns the amount of the dopants, the EDS evidenced Ba in an atomic percentage of 3.1% (Table 1), in very good agreement with the stoichiometric value of 3.4%. We were not able to detect Si with EDS, probably due to the overlapping of the Si peak (Kα = 1.74 keV) with the tail of the P peak (Kα = 2 keV). So, for the β-TCP-Si sample, the ICP-OES technique was employed. Values of Ca (100%), P (105%), and Si (106%) (n = 3, RSD% ≤ 10%) were detected, confirming the stoichiometry of the sample as well as the dopant presence.

All the characterisations performed on the β polymorph of Ca_3_(PO_4_)_2_ agreed on the effectiveness of the substitution of dopant elements. The β-TCP structure can admit dopant ions, at least in the small used amounts, without significant changes. The doping seemed instead slightly affect the external morphology, in particular by reducing the whole sample porosity.

#### 3.1.2. Cements

**Figure 4:**
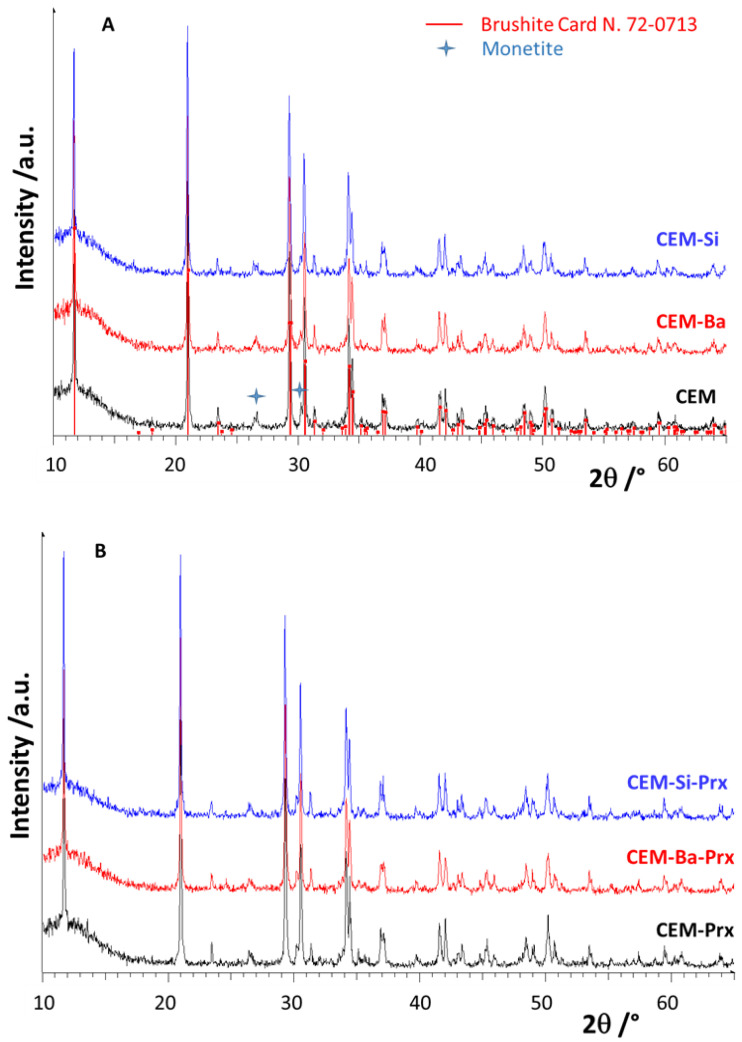
XRD patterns of pure and doped cements without (**A**) and with (**B**) piroxicam loading. The brushite red bars and the stars marking the main peaks of monetite are also reported.

The XRD patterns of pure and doped cements, without and with the piroxicam loading, are reported in Figure 4A and Figure 4B, respectively.

It is evident that all of them were constituted by the brushite phase, as demonstrated by the agreement with the expected peak positions of DCPD (red bars). The peaks of the drug were not evident (see also Figure S2, showing the XRD pattern of piroxicam), maybe due to its small amount, but also possibly to its transformation into an amorphous phase after the cement setting. Some small peaks due to monetite (CaHPO_4_), a common secondary phase for brushite cements [24], were evident (see the stars in Figure 4A). No peaks due to other calcium phosphates nor to residual of β-TCP were detected, demonstrating a complete dissolution-precipitation reaction. The drug presence did not hinder the cement formation.

**Figure 5:**
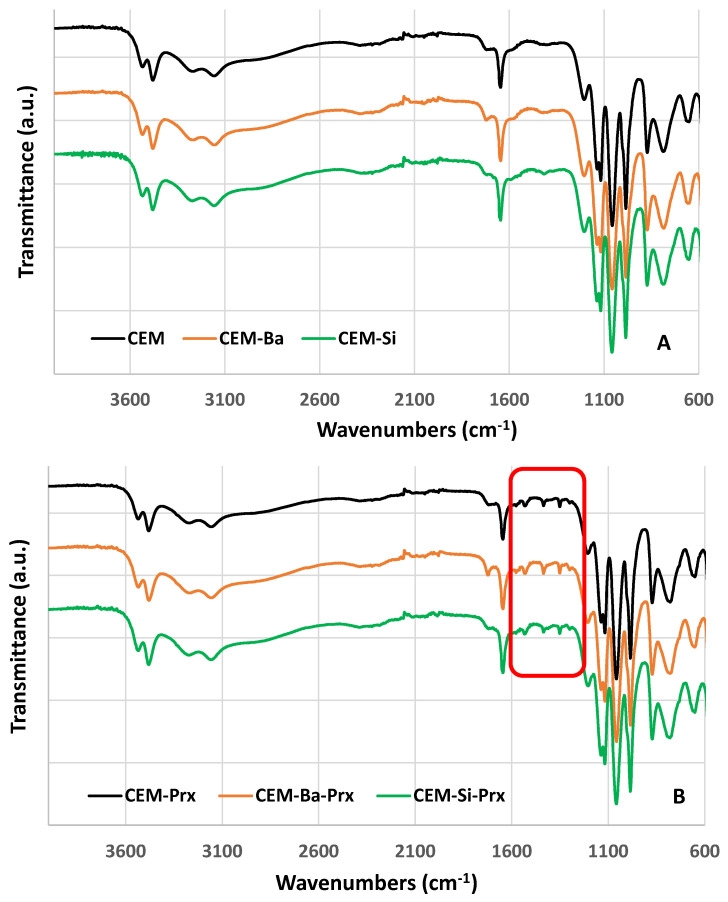
FT-IR spectra of pure and doped cements without drug (**A**) and loaded with piroxicam (**B**). The red box highlights the main drug peaks. The spectra were normalised considering the main peak of cement at about 1057 cm^−1^ as a reference (see text).

From the XRD analysis, the presence of the drug cannot be confirmed, as previously explained. However, by comparing the FT-IR spectra of pure and doped cements with those loaded with piroxicam, the peaks of the drug were well evident (Figure 5A,B), even if the most intense peaks in all the spectra were those expected for the brushite phase [24,25,26]. For a better comparison, the spectra were normalised by taking as a reference the main peak of cements at about 1057 cm^−1^.

The O-H stretching of water molecules is responsible for the bands located, for CEM, at 3536, 3478, 3278, and 3155 cm^–1^. The bands at 2388 (very broad) and 1645 cm^–1^ (very narrow) can be assigned to the O-H stretching of the hydrogen phosphate anions and bending of water molecules, respectively. The band at 1200 cm^–1^ was due to the in-plane P-O-H bending, while the P-O stretching was characterised by three strong bands at 1116, 1056, and 984 cm^–1^. The P-O(H) stretching and the evolution of water molecules gave rise to two bands at 871 and 778 cm^–1^, respectively. The band at 648 cm^−1^ is assigned to the P-O bending of the PO_4_^3−^ group [26].

No significant differences were evident between the pure cement and the doped ones (Figure 5A) [24,25]. In the CEM-Si spectrum, specific peaks due to Si-O bonds were unable to be evidenced, probably due to their overlapping with those of P-O bonds, but also to the small amount of substitution. In the FT-IR spectra of cements loaded with piroxicam, the drug vibrations were well evident, particularly in the 1600–1100 cm^−1^ spectral range, where the main absorptions of drug are expected (Figure 5B, red box and Figure S3 showing the FT-IR spectrum of piroxicam). The drug peaks can be seen in all the three cements, with the same intensities and positions, due to the addition of the same drug amount and to the same interactions with the inorganic brushite matrix. These peaks were slightly shifted to higher wavenumbers with respect to those of the pure drug, suggesting their involvement in electrostatic interactions between the brushite phase and piroxicam (see Figure S4).

**Figure 6:**
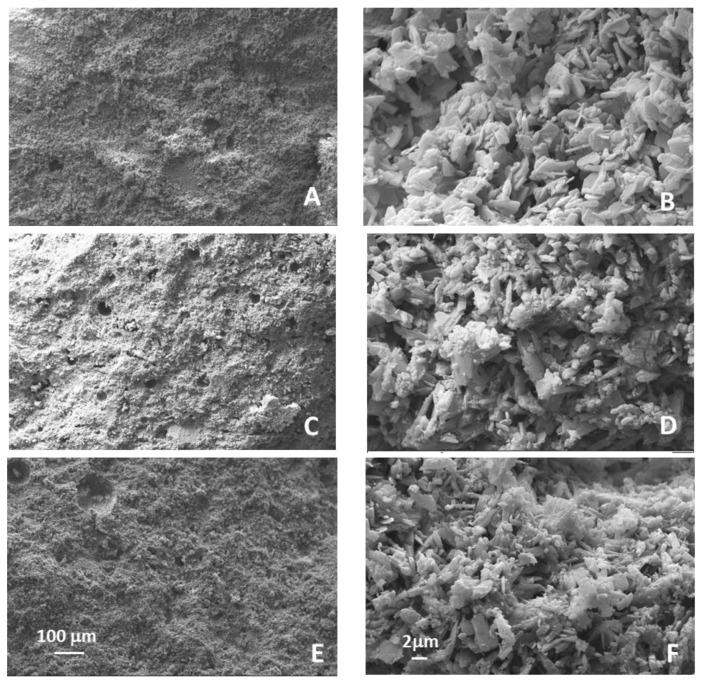
SEM images, at different magnifications, of cements without drugs: CEM (**A**,**B**), CEM-Ba (**C**,**D**), and CEM-Si (**E**,**F**). The magnifications are 300× (**A**,**C**,**E**) and 6000× (**B**,**D**,**F**). For the sake of simplicity, the bar of magnification is indicated in the (**E**,**F**) images and is valid for images at the same magnification.

The cement morphology was observed by SEM to evidence possible differences due to the dopant addition. In Figure 6, the images of pure and doped cements without drugs, taken on a part of the cement cylinders, are compared.

A good level of compactness was evident. The images with higher magnification made clear that the samples were constituted by flat grains with different dimensions, i.e., the typical morphology of the brushite phase [24,25,26]. No differences between CEM, CEM-Ba, and CEM-Si can be observed, suggesting that the dopant ions did not influence the external morphology, as also occurred for the crystal structure. So, notwithstanding the small differences in the external morphologies of pure and doped β-TCP, no effects were observed on the cements.

**Figure 7:**
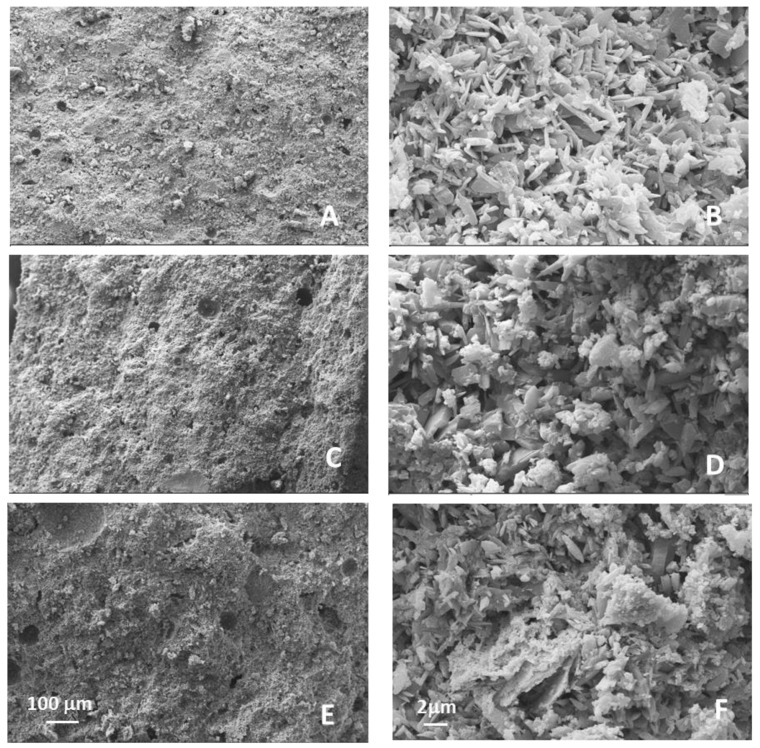
SEM images, at different magnifications, of cements with drugs: CEM-Prx (**A**,**B**), CEM-Ba-Prx (**C**,**D**), and CEM-Si-Prx (**E**,**F**). The magnifications are 300× (**A**,**C**,**E**) and 6000× (**B**,**D**,**F**). For the sake of simplicity, the bar of magnification is indicated in the (**E**,**F**) images and is valid for images at the same magnification.

The images were also taken on cements loaded with piroxicam to verify the possible effect of the drug on the cement forming morphology (Figure 7). The samples were highly compact, and the prevalent lamellar morphology was again evident, without differences between pure and doped cements. In the CEM-Si-Prx only, a fine component seemed present with respect to the other cements.

The EDS and ICP-OES analysis (on Si-doped cements) were also performed to verify the chemical compositions. The results are reported in Table 2. The Ca/P ratio was in good agreement with the stoichiometric ones, as well as the dopant amount (Ba and Si stoichiometric doping were 0.026 and 1.26 atomic %, respectively), within the standard deviation.

### 3.2. In Vitro Release

**Figure 8:**
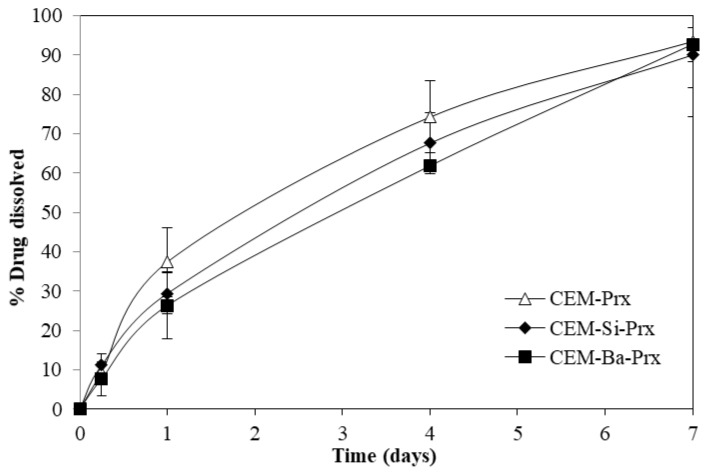
Dissolution profiles of Prx from the different samples, all containing 9 mg of the drug.

The dissolution profiles of the three cement samples clearly evidence the ability of the drug-loaded samples to sustain the Prx release without rate fluctuations (Figure 8). The dose is completely delivered in 7 days with a quite linear dissolution profile, as confirmed by the kinetics analysis (Table 3).

The zero-order model represents the best fitting for all samples with the higher correlation coefficient for CEM-Ba-Prx, followed by CEM-Si-Prx and CEM-Prx. The doping probably can allow for a better control of the release kinetics. The K_0_ values ranging from 0.5169 to 0.5378 corresponded to 0.046–0.048 mg of drug delivered per hour. Furthermore, the linear release profile of these samples was particularly interesting, being constant in the entire time range since, usually, the inert implantable matrices show substantially bimodal drug release profiles with an initial phase at a much higher speed, due to the dissolution of the drug present at the surface, followed by a second phase at a slower rate. It is instead critical from a therapeutic point of view that the drug becomes available at the site of action in a constant manner, avoiding fluctuations, for prolonged periods without at the same time exposing other organs that do not require the anti-inflammatory effect.

To evaluate the effect of doping and verify if the dissolution profiles can be considered similar or different, the *f*_2_ test was applied (Table 4). The results confirm that the two different types of cement doping gave rise to different dissolution profiles, suggesting that by suitable doping of the cements, the drug release rate could also be regulated.

### 3.3. Mechanical Measurements

**Figure 9:**
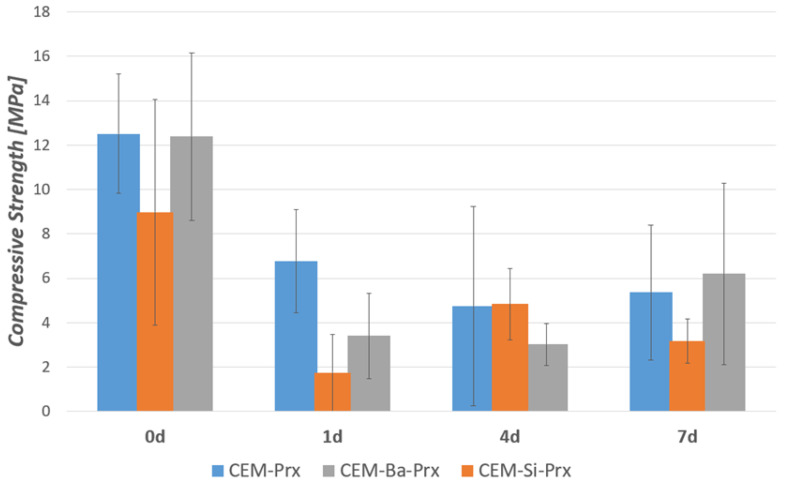
Histogram of the average compressive strength [MPa] values along with standard deviations at different times (see also Section 2.6 and Section 2.7).

**Figure 10:**
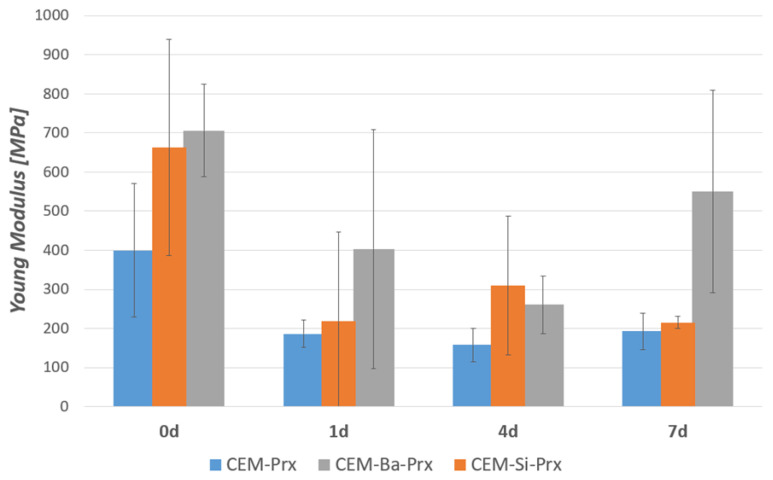
Histogram of the average Young’s modulus [MPa] values along with standard deviations at different times (see also Section 2.6 and Section 2.7).

Overall, both compressive strength and elastic modulus values exhibited a decreasing trend as the drug release duration increased, until reaching stable levels. An exception to this behaviour was observed in the CEM-Ba-Prx samples, which displayed higher values for both mechanical properties after 7 days of immersion compared to those recorded after 1 and 4 days of immersion, slightly lower than those measured in the not immersed condition. Notably, this behaviour mirrors what has been reported for strontium-doped brushite samples in previous studies (e.g., [24]). Furthermore, the CEM-Si-Prx samples immersed for 4 days showed a moderate increase in compressive strength and elastic modulus compared to other immersed samples. The undoped CEM-Prx samples exhibited the highest compressive strength values, even though CEM-Ba-Prx samples showed comparable measures, particularly in the not-immersed and 7-day-immersed conditions. Conversely, regarding the elastic modulus, the undoped samples displayed the lowest values, while the CEM-Ba-Prx samples consistently showed the highest values on average (Figure 9 and Figure 10 and Tables S2–S5).

Previous observations remain valid even considering the standard deviation results, which were still high compared to the average values. The high standard deviation was presumably due to the presence of voids in the structure of the samples, caused by air trapped during the forming process, as shown in Figure S5.

### 3.4. Post-Immersion Physical-Chemical Characterisation

**Figure 11:**
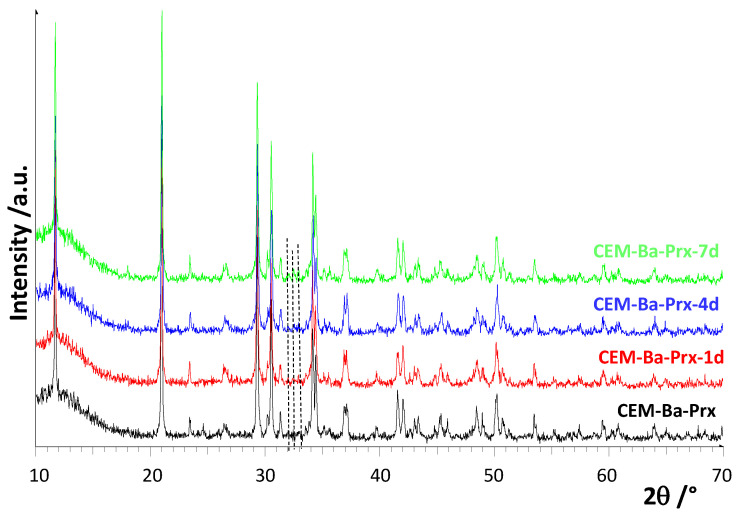
XRD patterns of Ba-doped cements after the immersion in phosphate buffer for different times, compared with the non-immersed cement. The dotted lines mark the positions of the main peaks of hydroxyapatite.

The same characterisation techniques previously discussed were also applied to the cements after the immersion in phosphate buffer for different times. A portion of the cement mould was ground in a mortar, and the powder diffraction patterns were collected. This analysis was useful to verify the possible cement structure evolution during the immersion and the consequent drug release. XRD patterns of CEM-Ba-Prx, chosen as an example, are reported in Figure 11, while those of CEM-Prx and CEM-Si-Prx are reported in Figures S6 and S7.

It is obvious that no changes in the peak positions and intensities can be seen for the Ba-doped samples, but also for the pure and Si-doped cements. This evidence suggests the stability of the brushite structure in the fluid, for at least up to 7 days. However, in carefully inspecting the patterns in Figure 11, new small peaks seemed to form between 31° and 33° (see the dotted lines in the figure). This was particularly evident after 7 days of immersion. These peaks can be attributed to the formation of hydroxyapatite, whose main peaks are located in this angular range [43,44]. This evidence was also present in the literature: the HAP formation can be attributed to the Ba^2+^ effect [29]. In fact, for pure and Si-doped cements, these peaks did not seem present, so suggesting that, in these cases, the conversion of brushite into HAP could be delayed in time.

**Figure 12:**
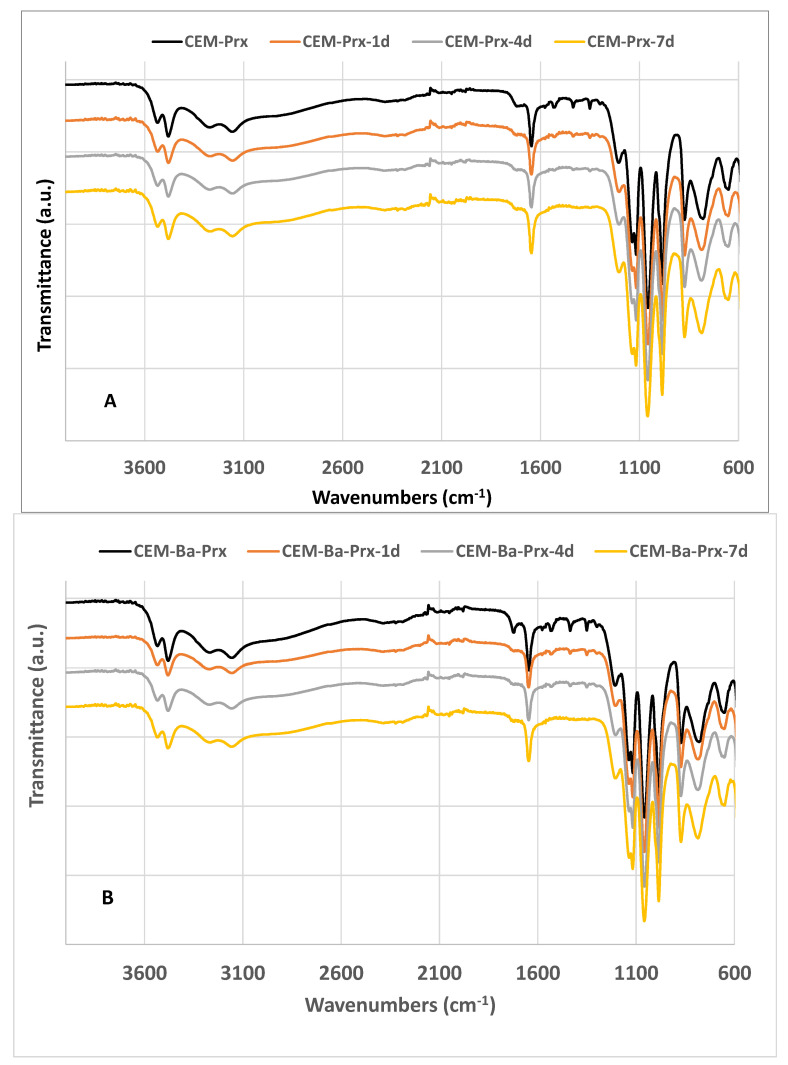

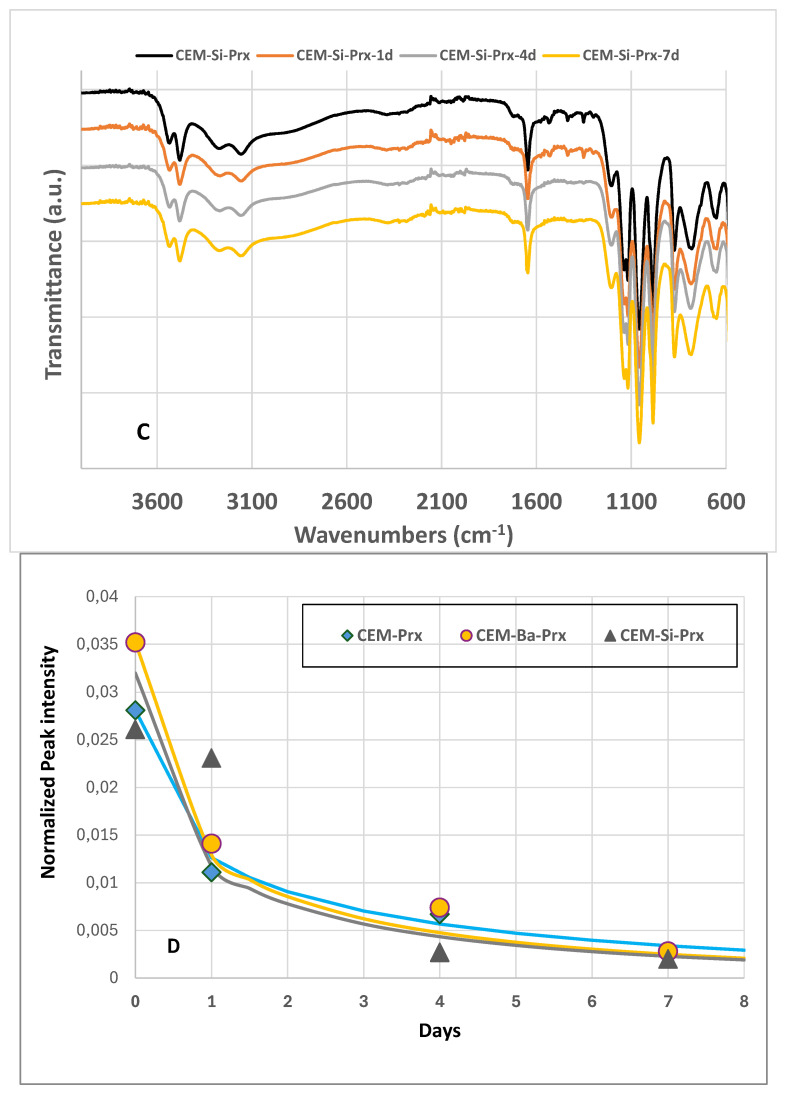
Normalised FT-IR spectra of pure (**A**) and Ba- (**B**) and Si- (**C**) doped cements after the immersion in phosphate buffer for different times. The normalisation was performed by taking as a reference the most intense peak of cement at about 1057 cm^−1^ (see also the text). In (**D**), the normalised intensity of the drug peak at about 1350 cm^−1^ is plotted versus the immersion time for the different cements. The lines are a guide for the eye.

The FT-IR spectra were also collected on the cements after the immersion in the fluid for different times. They are compared in Figure 12A–C together with that of the cement not immersed in solution.

**Figure 13:**
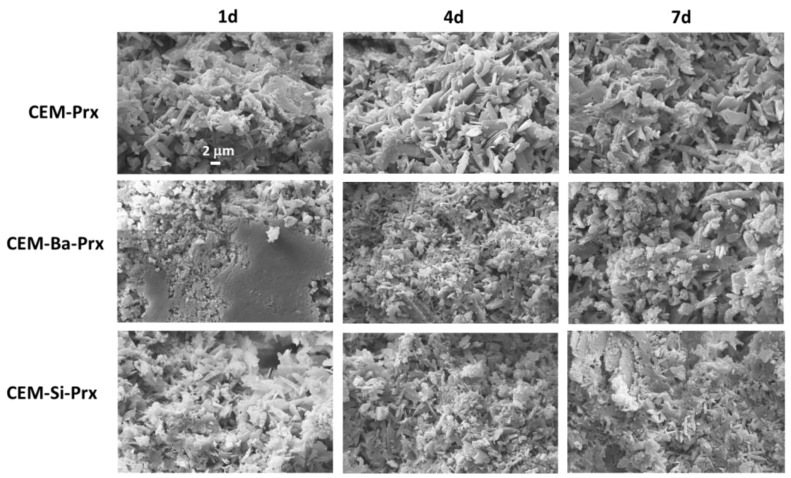
SEM images of the cement samples after different soaking times in the biological fluid. For all the images, the magnification is 6000×, and, for sake of simplicity, the bar with the enlargement is reported only in the first image.

Some observations can be made. For all the cements (pure and doped), it was observed that the main peaks of the drug (well evident in the spectral region 1600–1100 cm^−1^, see also Figure 5) tended to disappear by increasing the time of immersion, suggesting the drug release in the phosphate buffer, as clearly demonstrated by the dissolution study. The peaks decreasing can be well understood by inspecting Figure 12D, showing the trend of the normalised intensity of the drug peak at 1350 cm^−1^, chosen as an example, as a function of the immersion days. After 7 days, the peak practically disappeared, confirming the complete release of the drug (see also Section 3.2). The morphology evolution during the immersion time was also studied (Figure 13). The brushite cements maintained the flat form of grains, well evident for CEM-Prx and CEM-Si-Prx. For CEM-Si-Prx, the particle sizes seemed reduced and more aggregated with time. For Ba-doped cement, there was evidence of a fine component, whose amount seemed instead to reduce over time, with a consequent increase in the mean grain dimensions.

On the same samples, the EDS and ICP-OES analysis (for Si-doped samples) was performed to evaluate possible composition variations during the time of immersion. The results are reported in Table 5.

The Ca/P ratio of all the cements agreed well with the stoichiometric values within the measurement error (see also Table 2). This suggests that the chemical compositions were stable in the biological fluid, and no apparent loss of Ca or P seemed evident. Different was the case of Ba ions that, after 1 day of immersion, were clearly released in the fluid, because their amount was greatly reduced up to their complete disappearance after 4 days. This effect can be expected due to the high solubility of barium in similar biological fluids. Silicon too was released during this time, but more slowly with respect to Ba: after 7 days, an amount of about 60% was again present in the sample.

## 4. Discussion

The first step to obtain doped brushite cement is to find suitable substitutions to be performed in the β-TCP, one of the classical precursors of brushite cements [23]. We tested two dopant ions, with different properties, that can substitute calcium or phosphorous, in order to also verify which kind of substitution could be more effective on mechanical and pharmaceutical properties. Silicon, substituting phosphorous in the tetrahedra, was mainly chosen because it was reported that it could improve the mechanical properties and bone density of CPCs [30,31]. Barium, notwithstanding distrust, could substitute calcium in the crystallographic sites with higher coordination, and it could help in promoting hydroxyapatite formation, improving the osteogenesis and possibly mechanical properties [29]. It could become an interesting substituent when the scruples about its toxicity are solved by specific and detailed studies, which are currently missing.

The amount of dopant was chosen following examples taken from the recent literature [23,24,25]. In particular, for Ba doping, an amount of about 3 atomic % with respect to the Ca amount was used. We verified in fact that the increase in Ba amount could favour the formation of the monetite phase, as also demonstrated for Sr doping [23,24]. The presence of this phase, the anhydrous form of brushite, could vary the amount of water, which is a fundamental part of the setting reaction of cement enabling adjustments of the cement porosity, a determinant factor for the release kinetics of the loaded drug. So, we preferred to obtain single-phase brushite cements, and for this reason the dopant amount was maintained at a low level. For what concerns silicon, the chosen amount was 2.5 atomic % because a higher amount induced the formation of calcium silicates, so nullifying the aim to obtain Si doping of the β-TCP phase and of the corresponding brushite cement [30].

The substitution of both dopant ions in the β-TCP crystal structure was successful, and no variation of the crystalline structure was detected by X-Ray diffraction. The solid-state synthesis is suitable to perform doping, as long as the right reagents and temperatures are chosen. The presence of dopants introduced instead small variations in the external grain morphologies and in the vibrational modes of phosphate groups. In particular, the dopants seemed to reduce the sample porosity by improving the grains link and probably reducing the melting temperature, because the doped β-TCP seemed in some parts melted or near melting. The vibrational modes were the same as the pure β-TCP, but some of them were shifted to lower wavenumbers, maybe due to variations in the constant force of the bonds or the reduced mass due to the dopant presence. The brushite phases obtained starting from pure and doped β-TCP were also very similar: no significant variation in the crystalline structure of the cements was detected. This could be expected due to the small amount of dopant. However, from the vibrational spectra, a slight broadening of the bands of CEM-Ba can suggest some degree of structural disorder, in agreement with the findings on other doped cements [24,25]. This could be due to the different ionic radii of Ca^2+^ and Ba^2+^ ions (for an eightfold coordination Ca^2+^ 1.12 Å, while Ba^2+^ 1.42 Å), while a low effect is caused by silicon (for fourfold coordination Si 0.4 Å and P 0.31 Å). The external morphology was that expected for brushite, with flat grains, well evident for pure and doped cements.

To give the cements an anti-inflammatory activity, mainly useful in the early stages after surgery, we chose to load piroxicam into the cements during the brushite formation and to follow its dissolution characteristics. The drugs incorporated into brushite cement matrices must be carefully selected, as they need to be stable in acidic environments, due to the initial low pH of the cement; withstand the temperature changes during the setting reaction; and have appropriate adsorption to the cement [11]. We thought that piroxicam, never exploited to this aim in the bone cement field, could be a good choice.

Piroxicam, a highly crystalline drug, as demonstrated by its diffraction pattern (Figure S2), turned out to be amorphous when added to the cements, as occurred also for other drug delivery systems [43,44]. For this reason, from XRD, its presence cannot be demonstrated, but it was instead well evident from FT-IR spectra. The dissolution study demonstrated that all the cements were able to release the entire drug dose in 7 days in phosphate buffer, with a regular zero-order kinetic, i.e., the dissolution of the drug is a function of time only and proceeds independently of the concentration of the drug at any given time point. Drug release from cements is a complex phenomenon depending on different factors such as the matrix features (porosity, composition), the drug features (sizes and solubility), the presence of additives, the method of drug loading, and the drug amount and the powder/liquid ratio during cement preparation [8]. Some differences in drug release from the different cements were evidenced by applying the *f*_2_ test. In our case, due to the constant factors such as drug features, the loading of the drug in solid form, and the same ratio of 1 mL/2 g liquid/powder during CPC preparation, the small differences in the release curves could have been due to the different chemical compositions, i.e., the dopant presence. In particular, the most effective substitutions seemed to be those of barium, located on calcium sites, differently from silicon, substituting phosphorous. This can agree with the literature results that showed improved mechanical properties and injectability for Sr- and Mg-substituted cements, both dopants located on calcium sites [24,25]. It is expected that dopants with different ionic radii with respect to calcium can produce differences in bond force as well as distortion in the polyhedral coordination that can in turn influence the physical-chemical properties. Due to the high similarity of P and Si for what concerns the electronegativity and radius, a low impact of Si substitution on bonds and on the crystalline structure is expected.

Mechanical measurements exhibit distinct patterns, yet they also indicate that internal porosity within the specimens—stemming from bubble formation during preparation—results in defects, reflected in elevated standard deviation values. Nevertheless, an optimised process could address this issue. Enhancing the specimen structure can be achieved by using systems that exert a constant force during cement setting, thereby making the mixture more compact and devoid of air voids. Additionally, vacuum tools can be employed to remove any air presence within the material. Even so, the mechanical measurements show fairly clear trends, with both the compressive strength and the elastic modulus values decreasing and then stabilising as the immersion days increased. Specifically, the compressive strength was highest for the non-doped samples, while, conversely, their elastic modulus values were the lowest. This outlines a behaviour for the non-doped samples, characterised by higher compressive resistance, but lower stiffness. In contrast, the doped specimens exhibited a higher elastic modulus but reached lower compressive strength values, indicating a moderate fragile nature. This behaviour was particularly evident in the Ba-doped samples.

After the drug release, the cements maintained the original brushite structure as well as the external flat morphologies. The chemical compositions presented some intriguing trends. While the Ca and P amounts did not change during immersion, the Ba amount markedly decreased, yet after one day, and after 4 days, no barium was detected in the cement. This result is expected due to the high barium solubility in the biological fluid. The same evidence was observed for silicon, which was progressively released from the cements, even if more slowly with respect to barium. After one week, about 60% of silicon was again present in the sample. These observations suggest that the brushite-based CPCs can be appropriately designed not only for application in the orthopaedic field, being able to support relatively high loading, but also for drug delivery applications and the release of ions with specific functions for the body.

## 5. Conclusions

Brushite-based cements with application in orthopaedic implants are widely used but suffer from poor mechanical properties. It is particularly important to find new materials able to support high loading for prolonged times. In this context, doping with small amounts of different elements seems a promising way.

Pure and Ba- or Si-doped brushite cements loaded with piroxicam, an anti-inflammatory drug, were prepared by the dissolution-precipitation method to verify the dopant effect on the physical-chemical features, as well as on the drug release kinetics and mechanical properties. We chose Ba as a dopant never used in the literature and Si, which is rarely used in drug-loaded cements. The physical-chemical characterisation, based on the combination of different techniques, demonstrated the successful dopant insertion in the brushite structure, with the expected chemical compositions and the classical lamellar morphology of cements. The mechanical measurements showed fairly clear trends: both the compressive strength and the elastic modulus values decreased and then stabilised as the immersion days increased. Some problems due to a residual internal porosity, properly solved, will allow for reaching more clear trends. However, our most significant result is that the cements not only guaranteed a prolonged release of the loaded piroxicam amount, for at least 7 days, but also had constant release kinetics. This is a particularly important finding because our cements, differently from those reported in the literature, did not show the classical bimodal drug release profile with an initial phase at a much higher speed, due to the dissolution of the drug present at the surface, followed by a second phase at a slower rate. It can be suggested that by properly modifying the doping, i.e., the kind and amount, of the β-TCP and consequently of the cements and the way to obtain the cylinders, possibly avoiding defects, it would be possible to modulate further the dissolution rate of the drug, the mechanical properties, and the dopant ions released for specific functionalities.

The proposed cements could be useful for applications in the orthopaedic field, for drug delivery, and for the release of some necessary micronutrients to the body. Our results also show the importance of properly matching the results of different disciplines, in our case, chemistry, pharmacy, and engineering, to have a more complete knowledge of the materials and the possibility to better tune them for the different applications.

## Figures and Tables

**Table 1:** Elemental compositions (atomic percentage) determined by EDS microanalysis. The data are the average of the measurements in three different sample points.

	Ca	P	Dopant	Ca/P	% Dopant
β-TCP	17.9	11.22	-	1.59	
β-TCP-Ba	12.94	9.37	0.40	1.39	3.1
β-TCP-Si	14.06	8.39	-	1.67	-

(β-TCP = β-tricalcium phosphate; for the sample names, see Section 2.1).

**Table 2:** Elemental compositions of cements as determined by EDS (the results are the average of the measurements in different sample points) and ICP-OES for Si-doped samples (n = 3, RSD ≤ 10).

	Ca	P	Dopant	Ca/P	Ca/P Stoichiometric	Dopant %
EDS (atomic%)						
CEM	11.68	11.34	-	1.03	1	
CEM-Prx	10.05	10.10	-	0.99	1	
CEM-Ba	9.35	9.83	0.24	0.951	0.975	0.026
CEM-Ba-Prx	10.78	10.39	0.28	1.04	0.975	0.026
ICP-OES (% total)						
CEM-Si	103	102	100	1	1.012	1
CEM-Si-Prx	102	100	100	1	1.012	1

EDS = energy dispersive spectroscopy; CEM = cement; Prx = piroxicam; ICP-OES = inductively coupled plasma optical emission spectroscopy; for the sample names, see Section 2.2.

**Table 3:** Evaluation of the release kinetics using the zero-order and the first-order models. The correlation coefficients R^2^ and K_1_ values are reported.

	Zero-Order		First-Order	
	R^2^	K_0_	R^2^	K_1_
CEM-Prx	0.9199	0.5378	0.8296	0.0255
CEM-Si-Prx	0.9553	0.5169	0.9307	0.0109
CEM-Ba-Prx	0.9795	0.5348	0.8993	0.0109

(for the sample names, see Section 2.2).

**Table 4:** Evaluation of the similarity factor of the three dissolution profiles using the *f*_2_ tests.

Samples Compared with the *f*_2_ Test	CEM-Prx	CEM-Si-Prx
CEM-Si-Prx	59-Similar	-
CEM-Ba-Prx	49-Not similar	69-Similar

(for the sample names, see Section 2.2).

**Table 5:** Elemental compositions of cements determined after immersion for different soaking times in biological fluid (see text). For pure and Ba-doped samples, the data were derived from EDS measurements (average of the analysis in three different sample points) and are reported as atomic percentages, while for Si-doped cements, the data came from the ICP-OES analysis and are reported as average % of the total element amount (n = 3, RSD ≤ 10).

	Ca	P	Ca/P	Dopant
CEM-Prx-1d	10.38	10.62	0.98	
CEM-Prx-4d	11.56	11.20	1.03	
CEM-Prx-7d	11.10	10.91	1.02	
CEM-Ba-Prx-1d	10.42	10.91	0.955	0.09
CEM-Ba-Prx-4d	11.23	10.80	1.04	-
CEM-Ba-Prx-7d	10.42	10.91	0.955	-
ICP-OES	%	%		%
CEM-Si-Prx-1d	104	102	1	78
CEM-Si-Prx-4d	106	104	1	61
CEM-Si-Prx-7d	101	101	1	62

(for the sample names, see Section 2.2).

## Data Availability

The original contributions presented in this study are included in the article/Supplementary Materials. Further inquiries can be directed to the corresponding authors.

## Supplementary Materials

The following supporting information can be downloaded at https://www.mdpi.com/article/10.3390/ma18051065/s1. Figure S1. Compression test setup. Figure S2. XRD pattern of piroxicam. Figure S3. FT-IR spectrum of piroxicam. Figure S4. Comparison between the FT-IR spectra of loaded cements and that of piroxicam in the spectral range 1600–1100 cm^−1^ to evidence the drug peaks and their positions. Figure S5. Presence of voids in the tested specimens (see arrows). Figure S6. XRD patterns of pure cements loaded with piroxicam after different immersion time. Figure S7. XRD patterns of Si doped cements loaded with piroxicam after different immersion time. Table S1. Number of specimens tested for each type of cement for the mechanical measurements. For the sample names see Section 2.2. The letter d means days. Table S2. Average values of compressive strength [MPa]. For the sample names see Section 2.2. The letter d means days. Table S3. Average values of elastic modulus [MPa]. For the sample names see Section 2.2. The letter d means days. Table S4. Standard deviation values of compressive strength [MPa]. For the sample names see Section 2.2. The letter d means days. Table S5. Standard deviation values of elastic modulus [MPa]. For the sample names see Section 2.2. The letter d means days.
