# Supplementary material for: Pure and Doped Brushite Cements Loaded with Piroxicam for Prolonged and Constant Drug Release
Source: Materials (Basel). 2025 Feb 27;18(5):1065. doi: 10.3390/ma18051065 (PMC11901259; doi:10.3390/ma18051065)
Supplement: Supplementary file 1 [file materials-18-01065-s001.zip › materials-3456615-supplementary.pdf]

Supplementary file

Pure and doped brushite cements loaded with Piroxicam for prolonged and constant drug release

Marcella Bini, Giovanna Bruni, Michela Sturini, Beatrice Rossetti, Gianluca Alaimo, Ferdinando Auricchio, Valeria Friuli, Lauretta Maggi

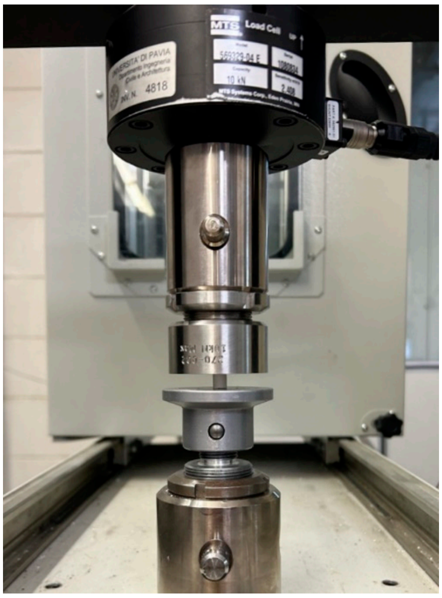

Figure S1 – Compression test set-up

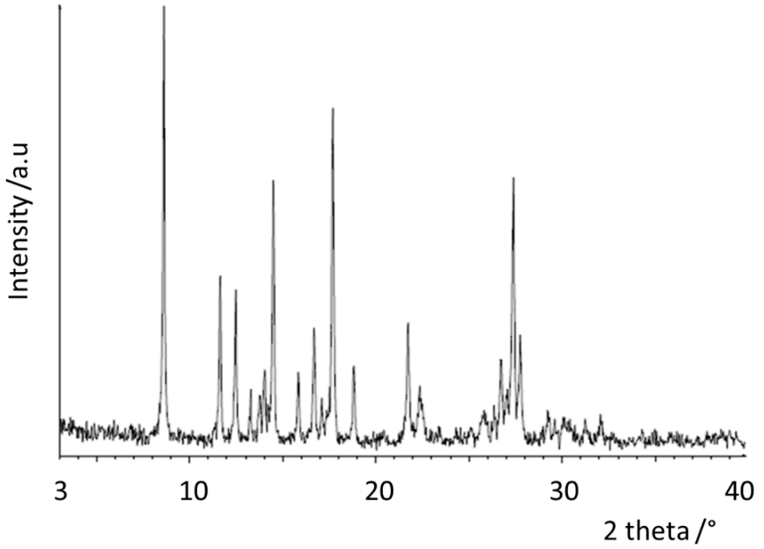

Figure S2 – XRD pattern of Piroxicam

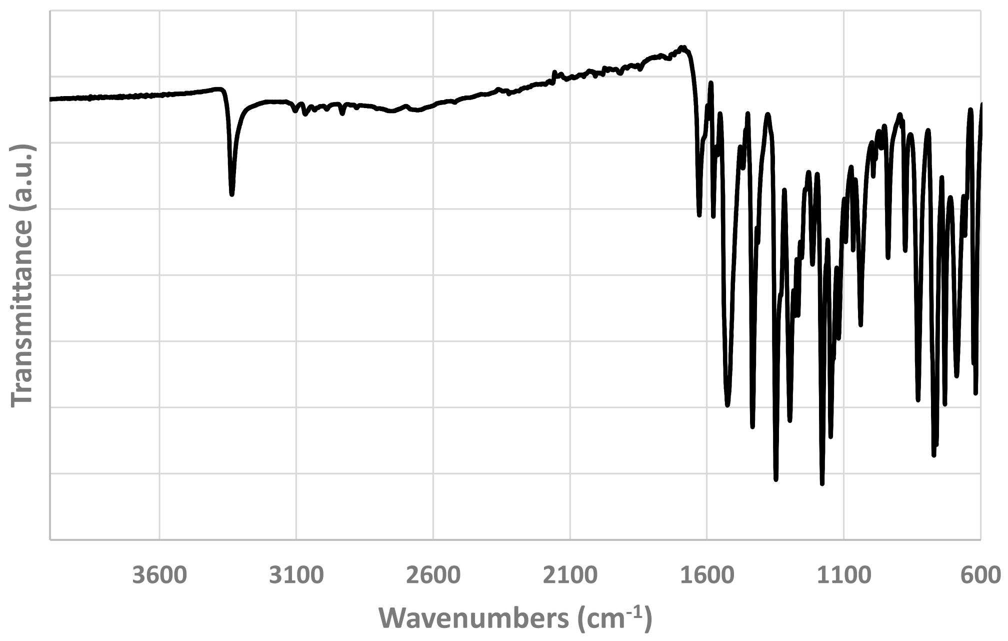

Figure S3 – FT-IR spectrum of piroxicam

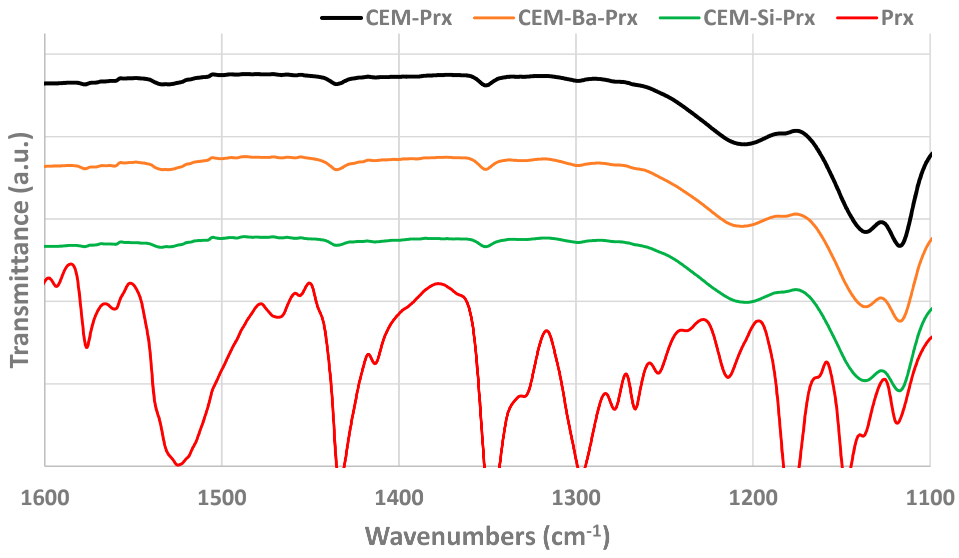

Figure S4 – Comparison between the FT-IR spectra of loaded cements and that of piroxicam in the spectral range 1600-1100 cm^{-1} to evidence the drug peaks and their positions.

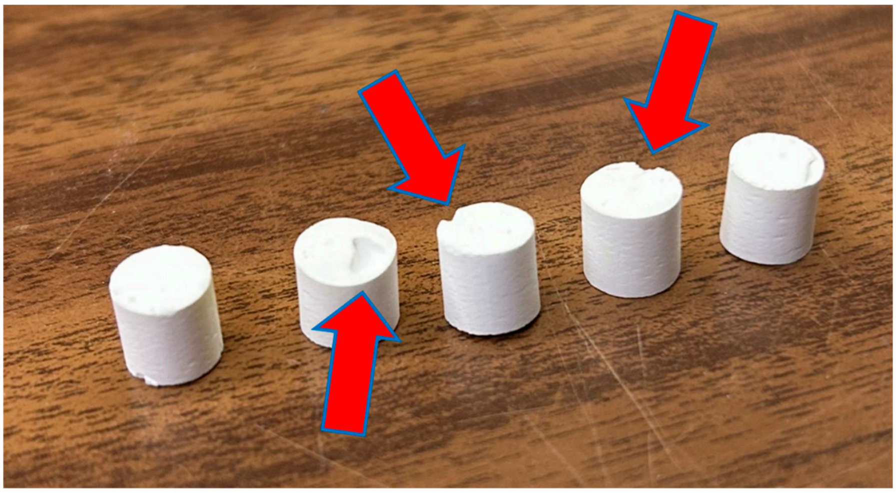

Figure S5 – Presence of voids in the tested specimens (see arrows)

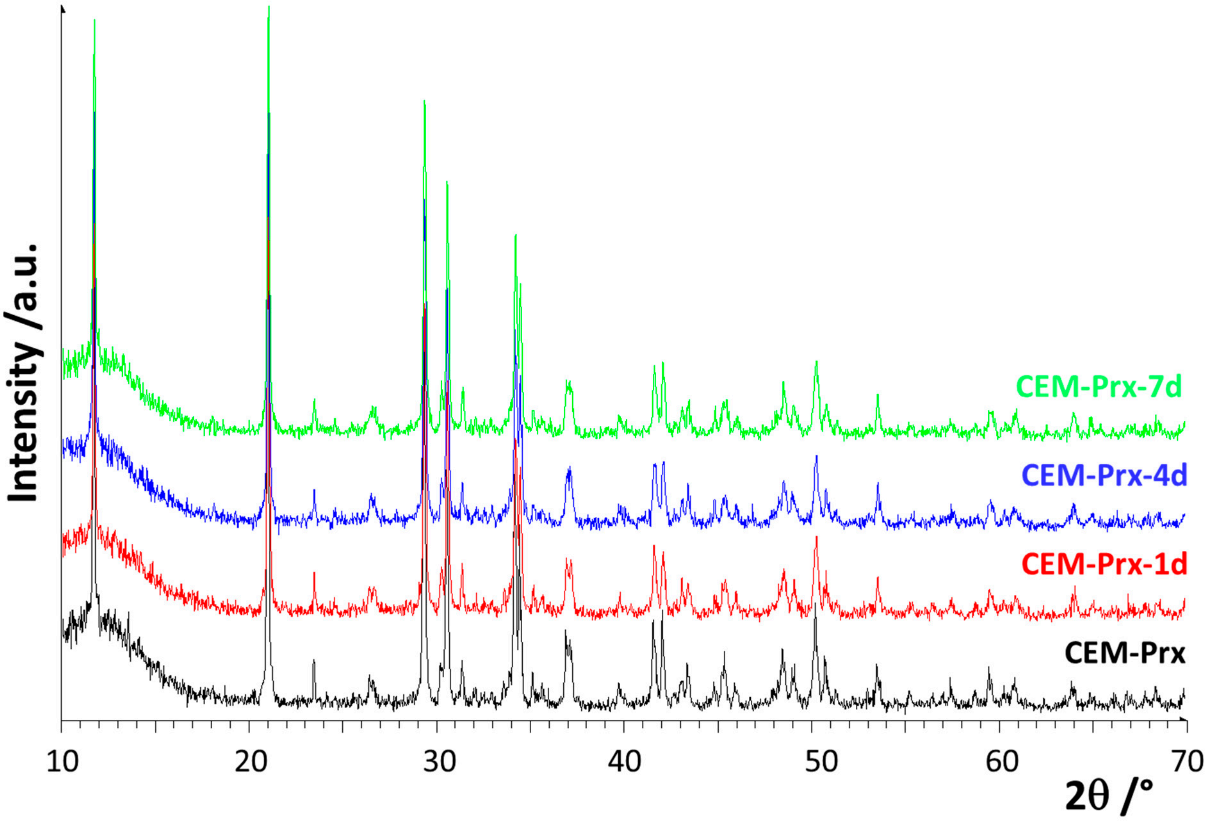

Figure S6 – XRD patterns of pure cements loaded with piroxicam after different immersion time.

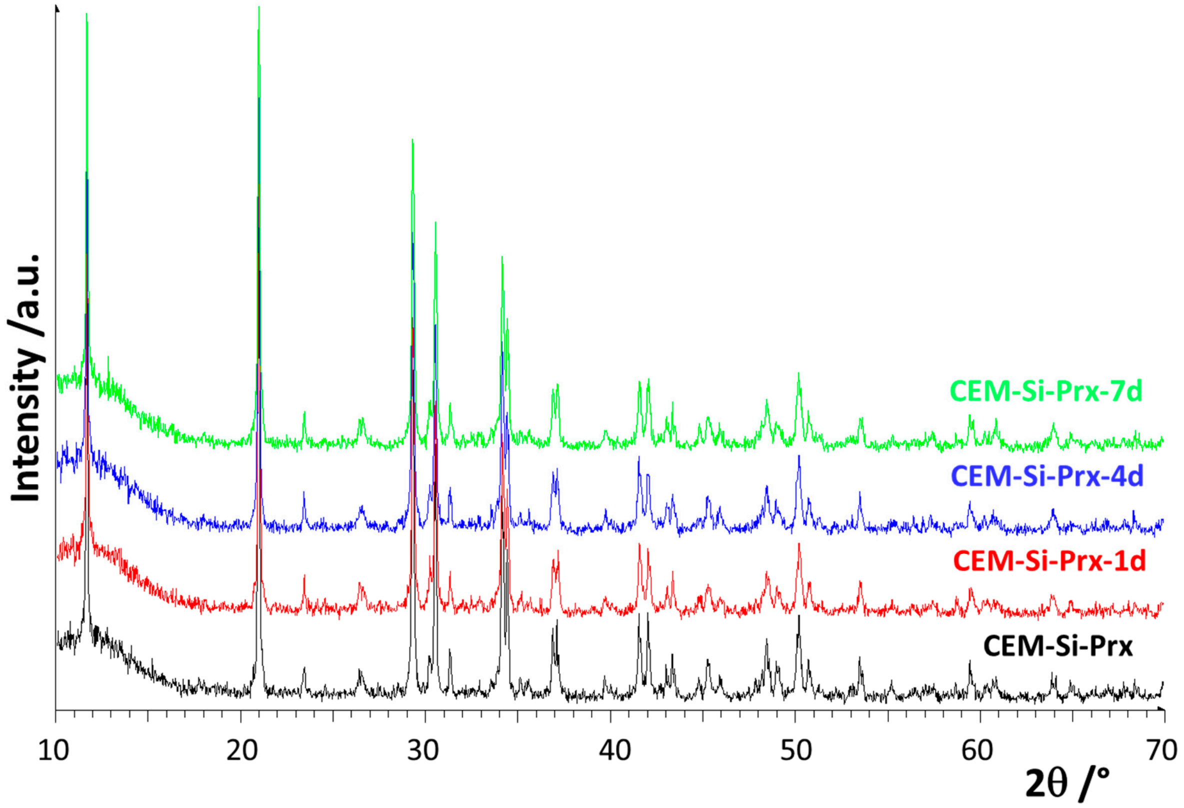

Figure S7 - XRD patterns of Si doped cements loaded with piroxicam after different immersion time.

	**0d**	**1d**	**4d**	**7d**
**CEM-Prx**	5	6	6	6
**CEM-Si-Prx**	3	3	3	3
**CEM-Ba-Prx**	4	3	3	3

Table S1 – Number of specimens tested for each type of cement for the mechanical measurements. For the sample names see section 2.2. The letter d means days.

	0d	1d	4d	7d
**CEM-Prx**	12,5	6,8	4,7	5,4
**CEM-Si-Prx**	9,0	1,8	4,8	3,2
**CEM-Ba-Prx**	12,4	3,4	3,0	6,2

Table S2 – Average values of compressive strength [MPa]. For the sample names see section 2.2. The letter d means days.

	0d	1d	4d	7d
**CEM-Prx**	399,9	186,5	157,6	192,5
**CEM-Si-Prx**	662,7	317,9	308,9	215,5
**CEM-Ba-Prx**	706,2	403,3	260,9	550,3

Table S3 – Average values of elastic modulus [MPa]. For the sample names see section 2.2. The letter d means days.

	0d	1d	4d	7d
**CEM-Prx**	2,7	2,3	4,5	3
**CEM-Si-Prx**	5,1	1,7	1,6	1
**CEM-Ba-Prx**	3,8	1,9	0,9	4,1

Table S4 – Standard deviation values of compressive strength [MPa]. For the sample names see section 2.2. The letter d means days.

	0d	1d	4d	7d
**CEM-Prx**	170,4	34,6	43	47
**CEM-Si-Prx**	276,8	229,6	177,5	15,6
**CEM-Ba-Prx**	118,4	306	73,5	260

Table S5 – Standard deviation values of elastic modulus [MPa]. For the sample names see section 2.2. The letter d means days.
